# An Interesting Case1Treated in Clinic of Dental Department, University of Pennsylvania.

**Published:** 1905-06

**Authors:** O. G. L. Lewis

**Affiliations:** Philadelphia


### AN INTERESTING CASE.[1]

BY O. G. L. LEWIS, D.D.S., PHILADELPHIA.

PATIENT, Marie Smith, aged fourteen years. Reported at the clinic April 7, 1904, for treatment of a growth which had developed between the upper central incisors. Growth was about the size of a common chestnut.

*History.*—Patient has a large birth-mark covering the entire right side of the nose and lip. This was first noticed, when a baby three days old, as a spot on the lip, and has gradually spread and grown until it is now beginning to show at various points on the left side of the nose. The growth for which treatment was desired was first noticed about one year ago as a small swelling. The gums at this time became very sensitive and bled upon the slightest provocation.

*Examination.*—On examination it was found that the birth-mark extended to the under surface of the lip, and that the gums were also more or less affected. There was found a marked condition of hypertrophy or hyperplasia along the entire free margin from the right lateral in front to the second molar behind. On the left side this condition was also found, but only to a limited degree. The large growth in front was found to be attached to the socket of the right central incisor; owing to its appearance and attachment and undoubted association with the disfigurement of the face it was diagnosed as an angiofibroma.

*Treatment.*—The teeth were carefully cleansed, considerable serumal tartar being removed from beneath the free margin of the gums. A mouth-wash of glycothymoline, fifty per cent. strength, to be used every two hours alternately with one of phenolsodique, three tablespoonfuls to the glass of water, was ordered. On April 15 the gums on the left side had assumed their normal condition, while a marked improvement was also noticed on the right side. At this sitting it was decided to remove the large tumor by means of strangulation. This was successfully accomplished, and at the next sitting the portions overhanging the lateral and cuspid were removed in the same manner. The rest of the mouth continues to improve slowly under weekly treatment. The gums are firm and

---

[1] Treated in Clinic of Dental Department, University of Pennsylvania.

natural-looking but are still considerably enlarged. Owing to the response that has been given to the treatment, I am inclined to believe that the condition, other than that of the large tumor, is due to hypertrophy and not to hyperplasia, and that the primary cause of the disturbance was the serumal deposits which were removed at the first sitting.

The prognosis for the general condition is favorable except in the case of the tumor between the centrals. If this was of partial or complete periosteal origin, and came from the socket of the tooth, it will undoubtedly return and may result in the subsequent exfoliation or extraction of that organ.

